# Evaluation of Simultaneous Dual-radioisotope SPECT Imaging Using ^18^F-fluorodeoxyglucose and ^99m^Tc-tetrofosmin

**DOI:** 10.7508/aojnmb.2016.02.002

**Published:** 2016

**Authors:** Yasuyuki Takahashi, Mizuki Mochiki, Keiko Koyama, Toshihiko Ino, Hiroyuki Yamaji, Atsuko Kawakami

**Affiliations:** 1 Department of Nuclear Medicine Technology, Gunma Prefectural College of Health Sciences, Maebashi, Japan; 2 Department of Radiological Technology, Gunma Cardiovascular Center, Maebashi, Japan; 3 Department of Diagnostic Radiology and Nuclear Medicine, Gunma Cardiovascular Center, Maebashi, Japan; 4 Department of Radiological Technology, National Kyushu Medical Center, Fukuoka, Japan; 5 Department of Radiological Technology, Maebashi Red Cross Hospital, Maebashi, Japan

**Keywords:** ^18^F-FDG, ^99m^Tc-tetrofosmin, DEW method, PET/SPECT system

## Abstract

**Objective(s):**

Use of a positron emission tomography (PET)/single-photon emission computed tomography (SPECT) system facilitates the simultaneous acquisition of images with fluorine-18 fluorodeoxyglucose (^18^F-FDG) and technetium (^99m^Tc)-tetrofosmin. However, ^18^F has a short half-life, and 511 keV Compton-scattered photons are detected in the ^99m^Tc energy window. Therefore, in this study, we aimed to investigate the consequences of these facts.

**Methods:**

The crosstalk correction for images in the ^99m^Tc energy window involved the dual energy window (DEW) subtraction method. In phantom studies, changes in the count of uniform parts in a phantom (due to attenuation from decay), signal detectability in the hot-rod part of the phantom, and the defect contrast ratio in a cardiac phantom were examined.

**Results:**

For ^18^F-FDG in the step-and-shoot mode, nearly a 9% difference was observed in the count of projection data between the start and end positions of acquisition in the uniform part of the phantom. Based on the findings, the detectability of 12 mm hot rods was relatively poor. In the continuous acquisition mode, the count difference was corrected, and detectability of the hot rods was improved. The crosstalk from ^18^F to the ^99m^Tc energy window was approximately 13%. In the cardiac phantom, the defect contrast in ^99m^Tc images from simultaneous dual-radionuclide acquisition was improved by approximately 9% after DEW correction; the contrast after correction was similar to acquisition with ^99m^Tc alone.

**Conclusion:**

Based on the findings, the continuous mode is useful for ^18^F-FDG acquisition, and DEW crosstalk correction is necessary for ^99m^Tc-tetrofosmin imaging.

## Introduction

In Japan, fluorine-18 fluorodeoxyglucose (^18^F-FDG) became commercially available in 2005, followed by the increased application of ^18^F-FDG positron emission tomography (PET) (1). ^18^F-FDG is employed for detecting cancer lesions and diagnosing cardiac sarcoidosis. The national health insurance system started to cover these applications in 2012. This background led to the considerable spread of PET evaluations at hospitals despite the absence of cyclotron or PET systems; so, a small number of PET evaluations are possible by using PET/single-photon emission computed tomography (SPECT) systems.

Approximately 250 similar PET/SPECT systems are installed around the world. A PET/SPECT system can employ two acquisition methods. One method applies septa for PET coincidence acquisition (2), while the other employs ultra-high-energy, high-resolution collimation (3-5) and allows simultaneous dual-radionuclide SPECT and PET acquisitions. Simultaneous acquisition has the advantage of acquiring two types of images with an exact temporal agreement.

So far, simultaneous dual-radionuclide acquisition has not been extensively studied. Therefore, this research aimed to examine the efficiency of simultaneous dual-radionuclide acquisition method through phantom studies by employing ^18^F-FDG and technetium (^99m^Tc)-tetrofosmin.

## Methods

### Acquisition and data processing conditions

The applied SPECT system was an Infinia 8+Hawkeye 4 (GE Healthcare, Milwaukee, WI, USA), equipped with ultra-high-energy, high-resolution collimators. The spatial resolution (full width at half maximum value) of this system was 8.6 mm at 140.5 keV and 11.8 mm at 511 keV. The rotation orbit was circular, the matrix size was 64×64, and the image reconstruction pixel size was 3.2×3.2 mm. The energy was set at 140.5±10.0% keV for ^99m^Tc and 511.0±7.5% keV for ^18^F. Xeleris (GE Healthcare, Milwaukee, WI, USA) was employed for data processing, and reconstruction was based on the implementation of the ordered subset expectation maximization algorithm (OSEM) (6, 7).

The number of iterations in OSEM was set at 10, and five subsets were used (7). A Butterworth filter (order 7, cut-off frequency=0.37 cycle/cm) was used as the pre-filter. The same pre-filter was used for both radionuclides, since myocardial accumulation of ^18^F-FDG is not constant; in fact, image quality is not fixed when the pre-filter changes in each case.

### Time attenuation effect

The ^18^F radionuclide has a short half-life of approximately 109 min, and over 20 min of acquisition, the count rate decreases by 12%. For SPECT, the step-and-shoot mode (one rotation at 6° intervals over 360°) or the continuous mode (four rotations) was employed (8). The two SPECT methods were performed within 20 min.

The cylindrical phantom (JSP type, Kyoto Kagaku Co., Ltd, Kyoto, Japan) was 20 cm in height and 20 cm in diameter. The phantom contained uniform and hot-rod sections. Within the hot-rod section, 15, 12, 10, 8, 6, and 4 mm diameter hot rods were placed in different sectors, which were located adjacent to one another. ^18^F, ^99m^Tc, or a combination of both radionuclides was injected into the phantom. The same activity concentration (45 kBq/ml) was applied for ^18^F and ^99m^Tc.

For calculating the reduced count rate over time, a region of interest was located over the uniform part of the projection data. In the projection count profile graph, the X-axis represented the position of projection data, while the Y-axis represented the standardized SPECT values. The effect of acquisition method was examined, using the hot-rod section of the phantom.

### Crosstalk effect

The crosstalk ratio in this study was analyzed by applying the data of the cardiac phantom (HLD type, Kyoto Kagaku Co., Ltd, Kyoto, Japan). In total, 1.8% of 740 MBq of ^99m^Tc-tetrofosmin was accumulated in the myocardium (9). However, the accumulation rate of ^18^F-FDG was not determined since it significantly varied in different cases. Therefore, the injection rate was set at 45 kBq/ml (nearly the same).

The crosstalk ratio was evaluated as the concentration of each radionuclide was changed. The cardiac phantom contained only a single radionuclide or both radionuclides, the concentration of the cardiac phantom with only ^99m^Tc was 20%, 40%, 60%, 80%, or 100% of 45 kBq/ml. In four measurements with both radionuclides, the concentrations of ^18^F and ^99m^Tc combination were 20%+80%, 40%+60%, 60%+40%, and 80%+20%, respectively.

After increasing the ^18^F count within the ^99m^Tc energy window (crosstalk), the DEW method was applied for crosstalk correction (4, 10). A window, centered at 140 keV (127-153 keV), was used for the primary photopeak events. The secondary window, bracketing 157 to 183 keV, corrected the scattered events.

The secondary window image was reconstructed, using events acquired in the lower window. Then it was subtracted from the pre-scatter correction image, reconstructed using events acquired in the photopeak window to produce the compensated (post-scatter correction) image. Count linearity in the ^99m^Tc energy window was examined after applying the DEW crosstalk correction.

The cardiac phantom was evaluated under two conditions, i.e., with and without a defect. The defect was located on the anterior wall (2 cm in size), and its detectability was compared under various conditions. ^18^F, ^99m^Tc, or both radionuclides were injected into the myocardium. With 45 kBq/ml of ^18^F and ^99m^Tc, the same volumes were injected into the myocardium.

As for the SPECT images, two types of quantitative analyses were conducted. By using the Bull’s eye map, with and without a defect, the radionuclide distribution was compared. The distribution of the radionuclide used the contrast ratio divided into 17 segments.

The SPECT values are expressed as mean ± standard deviation (SD). Data were analyzed, using EZR software (11). Repeated measures one-way ANOVA and least-square difference post hoc test were used for the comparison of three different images. The three different images were obtained by using ^99m^Tc as one of the dual radionuclides, for ^99m^Tc after crosstalk correction, and for ^99m^Tc imaged by itself, respectively. ^99m^Tc imaged by itself was standard. ^99m^Tc images were used to compare the contrast ratio (defect count/count of four directions around the defect) of the defect before and after DEW crosstalk correction. P-value less than 0.01 was considered statistically significant.

After completion of the phantom study, a patient was injected 740 MBq of ^99m^Tc-tetrofosmin at rest, and SPECT imaging was performed 30 min after the injection. Also, 185 MBq of ^18^F-FDG was administered to obtain simultaneous ^18^F-FDG and ^99m^Tc-tetrofosmin SPECT images.

## Results

**Figure 1:**
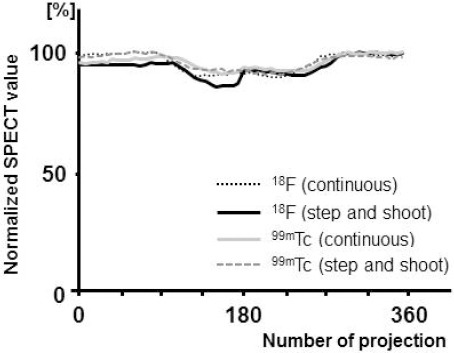
Count distribution of projection data. The continuous and step-and-shoot modes were applied in SPECT imaging

The projection count of ^18^F in the step-and-shoot mode showed discontinuity due to time attenuation between the start (0°) and end (180°) positions; the count difference was approximately 9% (Figure 1). On the other hand, in the continuous mode, ^18^F and ^99m^Tc had good continuity.

**Figure 2:**
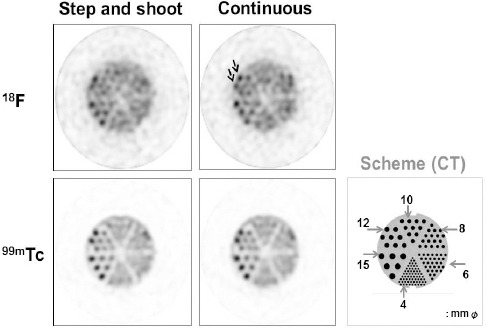
SPECT image of the hot-rod phantom (top: ^18^F, bottom: ^99m^Tc, left: step-and-shoot mode, right: continuous mode)

As for the hot-rod section of the phantom, ^18^F exhibited decreased visualization of 12 mm rods in the step-and-shoot mode, compared to the continuous mode (arrow in Figure 2). Visualization of 15 mm rods was confirmed at an equal distance in the continuous mode, whereas false images were obtained for hot rods, which were 10 mm or less in diameter in both acquisition modes. No difference was observed between the two modes in ^99m^Tc images.

**Figure 3:**
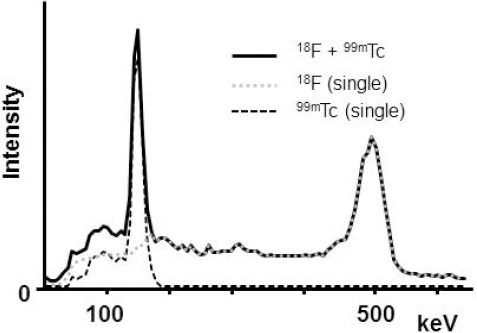
The energy spectra of ^18^F and ^99m^Tc by Infinia 8. The intensity was similar to that of the clinical study

**Figure 4:**
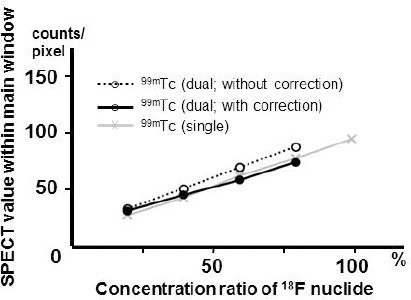
The count ratio of each single radionuclide and the combination of two radionuclides (the concentrations were changed). Single radionuclides were assessed with and without DEW crosstalk method. X-axis: concentration of ^99m^Tc as a single radionuclide (100%) and combination of two radionuclides (20%, 40%, 60%, and 80%). Y-axis: The count of the cardiac phantom per pixel

The energy spectra for each radionuclide and dual-radionuclide acquisition (with similar activities) are presented in Figure 3. The count of the ^99m^Tc energy window increased by 13% due to crosstalk from ^18^F. In the study of different concentrations, the count was measured in 20% increments from the 45 kBq/ml and a good linearity was detected in all cases (Figure 4). The regression equation was Y=1.05X+1.02 (R^2^=0.99) for ^99m^Tc alone and Y=0.88X+9.13 (R^2^=0.99) for ^99m^Tc from the dual-radionuclide acquisition. Although the increase in crosstalk from ^18^F concentration was clear, it was canceled by DEW crosstalk correction, and the regression equation was Y=0.91X+1.42 (R^2^=0.99).

In the visual evaluation of the cardiac phantom, Bull’s eye map of ^99m^Tc alone was similar to that of ^99m^Tc after crosstalk correction. Nearly the same number of divisions was reported for ^99m^Tc as one of the radionuclides in dual-radionuclide acquisition and ^99m^Tc after crosstalk correction.

**Figure 5:**
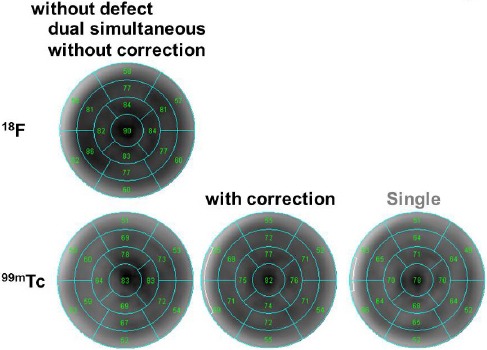
Bull’s eye map for the normal cardiac phantom without a defect for single and dual-radionuclide acquisitions

The normal phantom (without a defect) contained 14 segments, while the one with a defect had nine segments. As indicated in Figure 5, in the normal phantom, the SPECT values were 64.06±11.28, 67.06±9.72 (P<0.01), and 62.12±8.45 for ^99m^Tc as one of the radionuclides in dual-radionuclide acquisition, ^99m^Tc after crosstalk correction, and ^99m^Tc alone, respectively.

**Figure 6:**
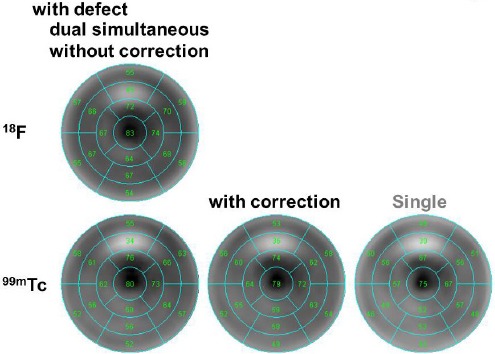
Bull’s eye map for the cardiac phantom, placed on the anterior defect for single and dual-radionuclide acquisitions

Defect detectability differed between ^99m^Tc alone and ^99m^Tc as one of the radionuclides in dual-radionuclide acquisition (Figure 6); however, the contrast of the defect was improved after DEW crosstalk correction. The contrast ratios were 14.46, 13.18, and 13.39 for ^99m^Tc as one of the radionuclides in dual-radionuclide acquisition, ^99m^Tc after crosstalk correction, and ^99m^Tc alone, respectively. The SPECT values were 60.18±10.51 (P<0.01), 59.65±10.26 (P<0.01), and 52.94±10.45 for ^99m^Tc as one of the radionuclides in dual-radionuclide acquisition, ^99m^Tc after crosstalk correction, and ^99m^Tc alone, respectively.

**Figure 7:**
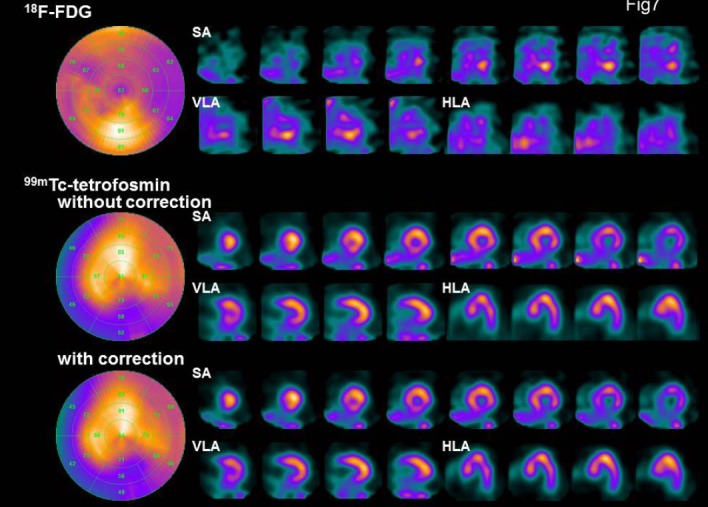
Bull’s eye map; SA: short axis, VLA: vertical long axis, and HLA: horizontal long axis in the evaluated patient. ^99m^Tc-tetrofosmin SPECT images were obtained before and after DEW crosstalk correction. An 81-year-old male was admitted to our center with chest pain. His risk factors for coronary artery disease included a prior history of hypertension and a family history of ischemic heart disease. The electrocardiogram showed ST-segment elevation in II, III, and AVF. The patient underwent selective left and right coronary artery angiography. The left coronary angiography showed moderate stenosis in segment VI. The right coronary angiography revealed 90% stenosis in segment I. He underwent stent placement in segment I. Seven days after stenting, simultaneous ^18^F-FDG and ^99m^Tc-tetrofosmin SPECT was performed

In the studied case (Figure 7), ^18^F-FDG imaging showed increased accumulation in the inferior wall from the base to the middle of the left ventricle; in addition, ^99m^Tc-tetrofosmin imaging showed decreased uptake in the mentioned area. In acute myocardial infarction, post-percutaneous coronary intervention of the revascularized area showed an increase in glucose metabolism and a decrease in myocardial perfusion.

The images with and without DEW crosstalk correction were compared. In the ^99m^Tc image after crosstalk correction, the contrast of the inferior-posterior wall was improved. In addition, the cardiac lumen was cleared in the short axis (SA) images.

## Discussion

In a SPECT system, the thickness of the Nal(Tl) scintillator is usually 3/8 of an inch; however, 511 keV photons of ^18^F can penetrate this thickness. In this study, the scintillators were 5/8 (5) or 1 inch thick in the PET/SPECT gamma camera. However, it should be noted that the spatial resolution decreases with increasing crystal thickness by one inch. Therefore, the StarBright Technology gamma camera (GE Healthcare, Milwaukee, WI, USA) uses a special procedure for the crystal to improve the intrinsic spatial resolution to 4.4-5.1 mm.

A PET/SPECT system can employ two acquisition methods. One method uses septa in PET coincidence acquisition (2) without utilizing SPECT, while the other employs ultra-high-energy, high-resolution collimation (3-5) and allows simultaneous dual-radionuclide SPECT and PET acquisitions, despite the reduced sensitivity of PET.

So far, simultaneous dual-isotope acquisition with ^18^F and ^99m^Tc has been only reported, using a standard gamma camera (3, 4, 5, 12). The motivation for this approach is that ^18^F-FDG can provide an image of myocardial glucose metabolism, while ^99m^Tc-tetrofosmin yields a myocardial perfusion image. ^18^F-FDG acquisition is also possible with simultaneous ^123^I-BMIPP acquisition, which can assess myocardial fatty acid metabolism (13). Therefore, simultaneous acquisition by dual radionuclides can provide two types of useful images, without a temporal change in the state of the patient. However, PET/SPECT imaging can improve upon the available system resolution with a gamma camera.

The influence of acquisition conditions on image quality has not been assessed in previous studies; therefore, we evaluated these factors in the present research. Based on the findings, time attenuation influenced the quality of ^18^F images. Therefore, we selected the continuous mode (8), which counteracted the influence of change in count density by repeated acquisitions. The step-and-shoot mode image appeared distorted, whereas the image improved in the continuous mode.

The energy spectrum showed that the accumulation rate in the assumed myocardium was similar in images acquired by ^18^F and ^99m^Tc, whether used independently or in combination. There was a 9% increase in the count rate by crosstalk from ^18^F to the ^99m^Tc energy window in comparison with ^99m^Tc alone. However, it should be noted that the accumulation rate of ^18^F in the myocardium differs, depending on the patient’s symptoms. Therefore, considering the concentration ratio, a cardiac phantom study was performed for ^18^F and ^99m^Tc.

Compton scattering in the ^99m^Tc energy window increased linearly. Even if there was a difference in terms of accumulation rate, the DEW crosstalk correction (4) was subtracted evenly. By this correction, the contrast of the part with a 2 cm defect clearly improved in the cardiac phantom study and provided a contrast equal to that of ^99m^Tc alone. Also, the contrast of the inferior-posterior wall improved for ^99m^Tc after crosstalk correction in our case.

In this study, we did not examine the ^18^F-FDG image in the coincidence mode. Therefore, we plan to compare the present findings (using collimators) with the results of coincidence mode in future.

## Conclusion

We investigated image improvement in PET/SPECT by using a gamma camera. The count rate, which decreased due to the short half-life of ^18^F, was improved by using the continuous mode. The crosstalk from the Compton-scattered photons of ^18^F into the ^99m^Tc window could be corrected, using the DEW method.
